# Bioactive, Antioxidant, and Nutritional Responses of Garlic (*Allium sativum* L.) to Fertilization Regimes

**DOI:** 10.3390/molecules31040652

**Published:** 2026-02-13

**Authors:** Boris Adamović, Jelena Visković, Aleksandra Tepić-Horecki, Anita Milić, Zdravko Šumić, Janko Červenski, Slobodan Vlajić, Snežana Jakšić, Milorad Živanov, Goran Jaćimović

**Affiliations:** 1 Faculty of Agriculture, University of Novi Sad, Trg Dositeja Obradovića 8, 21000 Novi Sad, Serbia; borisa@polj.uns.ac.rs (B.A.); goran.jacimovic@polj.uns.ac.rs (G.J.); 2 Faculty of Technology, University of Novi Sad, Bulevar cara Lazara 1, 21000 Novi Sad, Serbia; tepical@uns.ac.rs (A.T.-H.); anitavakula@uns.ac.rs (A.M.); sumic@uns.ac.rs (Z.Š.); 3 Institute of Field and Vegetable Crops, Maksima Gorkog 30, 21000 Novi Sad, Serbia; janko.cervenski@ifvcns.ns.ac.rs (J.Č.); slobodan.vlajic@ifvcns.ns.ac.rs (S.V.); snezana.jaksic@ifvcns.ns.ac.rs (S.J.); milorad.zivanov@ifvcns.ns.ac.rs (M.Ž.)

**Keywords:** garlic, fertilization treatments, bioactive compounds, antioxidant activity, phenolics, nutritional quality, functional food

## Abstract

Garlic (*Allium sativum* L.) is a significant crop cultivated worldwide for its culinary, nutritional, and medicinal value. This study aimed to evaluate the effects of different fertilization regimes on the bioactive compounds, antioxidant activity, nutritional value, and mineral composition of garlic. The field experiment was conducted at the Institute of Field and Vegetable Crops, in three replications. Fertilization significantly influenced the bioactive compounds, antioxidant activity, nutritional quality, and mineral composition of garlic. Cattle manure proved to be the most effective treatment, increasing protein (by approx. 5.1%), total sugars (17.9%), sucrose (24.9%), sulfur content (7.2%), total phenolics (3.1%), flavonoids (30.7%), and antioxidant activity (by 5.2–23.1% depending on the assay) compared to the control, indicating superior nutritional and functional quality. Multivariate analyses highlighted the significant impact of fertilization regimes on garlic quality, with mineral fertilizer, control (treatment without fertilizer application), and cattle manure enhancing bioactive compounds, antioxidant activity, and nutritional composition. Fertilization had limited effects on macroelements, although cattle and sheep manure increased nitrogen and sulphur contents, while molasses reduced phosphorus and potassium levels. Organic fertilization significantly modified microelement composition, with sheep manure notably increasing zinc and copper, while most fertilizers reduced boron, iron, and sodium contents compared with the control. Animal-based fertilizers, particularly cattle manure, provide a sustainable alternative to mineral fertilization, enhancing garlic’s dry matter, nutritional and bioactive compounds, and antioxidant activity.

## 1. Introduction

Garlic (*Allium sativum* L.) is a globally significant crop valued for its culinary, nutritional, and medicinal properties. As one of the most widely consumed *Allium* species, it presents a key role in diverse cuisines and is particularly appreciated for its characteristic flavor and aroma that result from its rich content of organosulfur compounds [1]. Beyond its culinary importance, garlic has long been recognized for a wide range of health-promoting effects, including anticancer, antioxidant, antidiabetic, cardioprotective, antibacterial, and antifungal activities [2,3]. Garlic bulbs are abundant in sulfur-containing phytochemicals such as alliin, allicin, ajoenes, vinyldithiins, and flavonoids, including quercetin [4]. Garlic bulbs are mostly composed of water and carbohydrates, which constitute around 65% of their fresh weight and 77% of their dry weight, respectively [5,6]. The water-soluble, high-molecular-weight fructose polymers known as fructans compose the majority of carbohydrates [7]. Proteins, pectin, minerals, and polyamines additionally exist in garlic cloves [8]. Proteins constitute approximately 3–7% of the dry weight and include enzymes crucial for sulfur metabolism, most notably alliinase, which catalyzes the formation of allicin and related organosulfur compounds central to garlic’s aroma and bioactivity [1]. In addition, garlic contains pectins, lipids in small quantities, and a wide range of mineral elements such as potassium, phosphorus, sulfur, calcium, magnesium, zinc, iron, manganese, copper, and selenium, and all of them support key metabolic and physiological processes [9].

The composition and concentration of these biochemical constituents are strongly influenced by soil nutrient availability and agronomic management. Proper fertilization management is essential for achieving high garlic yield, optimal nutritional quality, and enhanced bioactive compound content [10,11]. Efficient fertilization ensures that plants receive adequate nutrients at the right time, while supporting sustainable cultivation and minimizing environmental impact [12]. Organic fertilizers, such as cattle and sheep manure, provide a viable alternative to conventional mineral fertilizers by maintaining soil health and promoting the production of garlic with superior functional and nutritional properties [12,13,14]. Adequate nitrogen, phosphorus, and potassium levels enhance carbohydrate accumulation, bulb formation, protein synthesis, and both yield and nutritional value. Nitrogen supply plays a key role in regulating the sulfur assimilation pathways that govern the biosynthesis of alliin and related organosulfur compounds, while sufficient sulfur availability further supports the formation of sulfur-containing amino acids and volatile sulfur metabolites that define garlic’s characteristic aroma, flavor, and medicinal value [10,11].

Sustainable fertilization strategies have gained increasing importance in recent decades due to the environmental concerns associated with intensive mineral fertilizer use, such as soil degradation, loss of organic matter, and nutrient leaching. Organic fertilizers—including manures, composts, and agro-industrial by-products—provide a range of agronomic benefits. They improve soil structure, increase soil organic carbon, enhance microbial activity, and provide slow and steady nutrient release [12]. Such sustained nutrient availability is particularly beneficial for garlic, which requires a continuous and balanced nutrient supply during bulb development. Several studies have demonstrated that the application of manures, composts, and other organic fertilizers can significantly increase garlic yield and improve its nutritional and phytochemical properties, including phenolic content and antioxidant capacity [13,14].

Global garlic production has expanded substantially in recent years, driven by increasing consumer demand, the crop’s adaptability to diverse agro-ecological environments, and its economic importance in both commercial and smallholder farming systems [15]. Understanding how different fertilization strategies influence garlic quality, biochemical composition, and productivity is therefore crucial for optimizing cultivation practices and meeting growing market expectations. The main objective of the present research was to provide a comprehensive assessment of the bioactive compound content and antioxidant activity of garlic produced in Serbia under different fertilization regimes. Treatments included mineral fertilizer, poultry manure, cattle manure, sheep manure, supercompost, and molasses. The analyzed parameters comprised total dry matter, total phenolic and flavonoid compounds, antioxidant activity, proteins, lipids, sugars, and mineral elements. Additionally, principal component analysis (PCA) and cluster analysis were performed to identify relationships between production conditions and the biochemical characteristics of garlic samples.

Optimizing fertilization is essential for maximizing both the yield and quality of garlic. While the majority of studies have focused on mineral fertilizers, organic inputs such as cattle and sheep manure provide a sustainable alternative that can enhance nutritional value, increase bioactive compound content, and improve antioxidant activity. The present study demonstrates that field application of organic fertilizers can effectively enhance garlic quality without substantial additional planting costs, offering practical guidance for sustainable cultivation. Nonetheless, this research has certain limitations, including its single-location, small-scale design and the lack of long-term assessment of soil and crop responses. Future investigations should consider multi-location trials, evaluate the long-term effects of organic fertilization on soil fertility and crop productivity, and optimize combinations of organic inputs to further improve garlic quality and yield, thereby supporting the development of cost-effective and sustainable garlic production systems.

## 2. Results and Discussion

### 2.1. Bioactive Compounds and Antioxidant Activity of Garlic

Considering the recognized role of phenolic compounds in human nutrition and their beneficial effects, the content of these compounds has been extensively studied in various vegetable species and varieties. Flavonoids are recognized for their roles in slowing aging, reducing inflammation, protecting the nervous system, and supporting overall health [16,17].

The average phenolic content in this study (133.11 mg/100 g DW) (Table 1) was higher than the values reported for garlic [18,19]. In a study on celery, similar values were reported in celery root, while the phenolic content in celery leaves was significantly higher [20]. The highest phenolic content in garlic was recorded in the cattle manure treatment (155.57 mg/100 g DW), although this was not significantly different from the control (treatment without any fertilizer application) and mineral fertilizer treatments (Table 1). In the other organic fertilizer treatments, phenolic content was significantly lower. Higher phenolic content has been found in garlic, yellow onion, red onion, and leek [18], as well as in carrot and lettuce [21], when grown under organic systems compared to conventional systems. In celery root, the highest phenolic content was measured in the control, although it did not differ significantly from the other organic and mineral fertilization treatments. Fertilization decreased phenolic content in cabbage [22] and rapeseed [23], while it increased phenolic content in Welsh onion [24] and carrot [25]. Based on the results of this study, it can be concluded that garlic with a high phenolic content can be successfully grown using cattle manure.

The total flavonoid content (TF) in the analyzed garlic samples ranged from 20.01 to 36.21 mg CE kg^−1^ DW, with a mean value of 28.58 mg CE kg^−1^ DW (Table 1). The lowest TF was observed in the molasses treatment, while the highest TF was found in the supercompost treatment. There was no significant difference between the supercompost and cattle manure treatments. The variation in flavonoid content can explain differences in nutrient availability among the tested treatments [20]. Nutrient sources for plants mineralize at different rates, depending on the type of fertilizer, the degree of organic matter decomposition, temperature, and microbial activity, and their effectiveness as plant nutrient sources can therefore vary considerably [26]. The average flavonoid content in this study was higher than the values reported for garlic [19,27,28,29], while it was comparable to the values reported by Park and Kim [30]. Flavonoid content varies depending on the plant species as well as the variety [27]. The root and leaves of celery contain higher flavonoid content than garlic [20,31,32], as do tomato, pumpkin, red onion, leek, shallot, red lettuce, and watermelon [31].

*Allium* species exhibit different antioxidant activities (AA) under various conditions [24]. In the present study, the AA measured by the DPPH assay in the garlic samples ranged from 0.08 to 0.15 mg/g DW, with a mean value of 0.12 mg/g DW. The highest AA was recorded in the cattle manure treatment, while the lowest value was achieved in the molasses treatment (Table 1). Compared with cattle manure, no significant differences were observed between the control, mineral fertilizer, sheep manure, and supercompost treatments. The application of mineral fertilizer reduced AA by 7.1% compared to the control, which contrasts with the findings of Zhao et al. [24], who reported an increase in AA in Welsh onion following mineral fertilizer application. The application of poultry manure and molasses resulted in reduced AA compared to all other treatments. Garlic exhibited 23.8% higher AA when produced under organic conditions compared to conventional production systems [18].

Antioxidant activity measured by the FRAP assay ranged from 0.09 to 0.16 mg Fe^2+^/g DW. The lowest value was observed in the molasses treatment, while the highest AA (FRAP) was determined in the cattle manure treatment. Application of cattle manure increased AA by 23.07% compared to the control, whereas the remaining organic fertilizers reduced AA (Table 1). No significant difference was found between the control and sheep manure treatments. Mineral fertilizer reduced AA by 15.4% compared to the control, which aligns with the findings for cabbage [22]. Garlic, yellow onion, red onion, and leek showed higher antioxidant activity when grown under organic production systems compared with conventional systems [18], whereas Nicoletto et al. [22] reported lower AA in cabbage and lettuce when fertilized with organic fertilizers or a combination of organic and mineral fertilizers. Antioxidant activity depends on plant species, variety, and plant organ [18,20,27,32].

The range of AA measured by the ABTS assay in the garlic samples was between 0.44 and 0.61 mg/g DW, with a mean value of 0.52 mg/g DW. The application of cattle manure significantly increased AA compared to the control, while all other organic fertilizers significantly reduced AA (Table 1). Similarly, mineral fertilizer reduced AA by 6.9% relative to the control, which contradicts the findings of Zhao et al. [24]. All three assays showed the highest AA in the cattle manure treatment, where high flavonoid content was also recorded. This is consistent with Pietta [16], who states that flavonoids possess strong antioxidant properties. The reduced AA measured by the DPPH, FRAP, and ABTS assays in certain garlic samples may be attributed to lower phenolic content. This decrease is significant, as total phenolics are recognized for their strong antioxidant properties and contribute substantially to the overall antioxidant capacity of a plant [33].

### 2.2. Nutritional Composition of Garlic

The dry weight (DW) content is an important indicator of garlic bulb quality. In addition to its use as a fresh vegetable, garlic is an important raw material for the processing industry, where it is commonly dehydrated for incorporation into various spices. Higher DW content increases the yield of dried garlic per unit of fresh bulb. Furthermore, bulbs with higher DW content require less energy for the drying process. The average DW content observed in this study (31.47%; Table 2) was comparable to the values reported by other researchers [18,34]. Among the treatments, the application of molasses resulted in the highest DW content (33.23%), while all other treatments achieved significantly lower DW contents. The sheep manure treatment showed the lowest DW content (30.58%), which was significantly lower than the other treatments, except mineral fertilizer (Table 2). These results are in accordance with findings for leek [35], as well as for celery root [20], but in contrast with the results for garlic [18], where significantly higher DW content was recorded in garlic cultivated conventionally using mineral fertilizers. An increase in dry matter content in garlic fertilized with organic fertilizers compared to controls has also been reported [36]. Among treatments with organic fertilizers of animal origin, poultry manure achieved significantly higher DW content (31.81%) compared to cattle and sheep manure. Molasses and supercompost, as by-products of the food industry, had different effects on the DW content of garlic. Higher dry matter content in celery roots and leaves under supercompost treatment, without a statistically significant difference compared to molasses, has been reported [20]. DW content also affects storage potential, as bulbs with higher DW content can be stored for longer periods, since higher levels of DW reduce water loss during storage.

The protein content in this study varied from 5.14% to 5.77%, with an average value of 5.43% (Table 2). Relatively low protein content, ranging from 2.6% to 3.0% depending on garlic variety and cultivation practices, has been reported [37]. Comparable protein content values were documented by Eissa et al. [38]. In contrast, Sajid et al. [39] reported a crude protein content of 7.87%, whereas Czech et al. [18] reported values ranging from 15.03% to 17.14%. Fertilization with cattle manure (5.77%) and molasses (5.14%) significantly affected the protein content of garlic compared to the control. Application of cattle manure resulted in an increase of 0.28%, while molasses treatment caused a 0.35% decrease compared to the control. Application of mineral fertilizer and cattle manure increased protein content compared to the control, whereas all other treatments resulted in lower protein content (Table 3). These results are in agreement with Yatsenko et al. [36], who reported an increase in garlic bulb protein content following the application of organic fertilizers compared to the unfertilized control. Higher protein content has been found in garlic and leek grown under conventional systems, whereas yellow and red onions have higher protein content under organic systems [18]. Similarly, higher protein content in garlic, carrot, tomato, and okra grown conventionally compared to through organic cultivation has been observed [40]. In the present study, the molasses treatment resulted in the lowest protein content. This finding aligns with the observation that celery root exhibits the lowest protein content following fertilization with molasses [20].

The total fat content ranged from 0.08% (supercompost) to 0.24% (control and mineral fertilizer), with an average value of 0.17% (Table 2) which is lower than the values reported by other researchers [38,39]. The application of organic fertilizers significantly reduced fat content, whereas the use of mineral fertilizer had no effect, as fat levels in the control and mineral fertilizer treatments were identical. These results are not consistent with reports of an increase in fat content in garlic following the application of organic fertilizers [34,36]. The supercompost treatment resulted in the lowest fat content, consistent with findings in celery root fertilized with supercompost [20].

A statistically significant variation (CV = 36.89%) in total sugar content was observed among the garlic samples, ranging from 7.83% (supercompost) to 20.62% (cattle manure) (Table 2). The highest sugar content was achieved in the treatment with cattle manure. The highest total sugar content in celery root has also been reported with cattle manure treatment [20]. The supercompost treatment had the lowest total sugar content, which contrasts with previous findings reporting the lowest total sugar content in the no-fertilizer treatment [34]. No significant differences were observed between the control and mineral fertilizer treatments, or between sheep and poultry manure treatments (Table 4). Cui et al. [41] reported that higher sugar content was observed in treatments with lower doses of N, P_2_O_5_, and K_2_O. All organic manure treatments, except for cattle manure, resulted in lower total sugar content than the mineral fertilizer treatment. This finding contrasts with Golubkina et al. [35], who reported higher total sugar content in leeks grown under organic systems compared to conventional systems, as well as with Yatsenko et al. [36], who observed an increase in total sugar content in garlic bulbs following the application of organic fertilizers.

Fertilization had a significant effect on the sucrose content in garlic (*p* < 0.001; CV = 54.60%). Application of cattle manure (15.71%) had the highest effect and increased sucrose content by 3.13% compared to the control, and this increase was statistically significant. Other organic fertilizer treatments, as well as mineral fertilizer, supercompost and molasses, resulted in a significant decrease in sucrose content (Table 2). In contrast, the application of organic fertilizers was reported to increase sucrose content in garlic bulbs [36]. Most studies on garlic do not report sugar content, with particularly limited data on sucrose [42]. The same authors reported sucrose content ranging from 1.99 to 3.08 g/100 g DW, with variations depending on the variety and cultivation location. The values obtained in this study were higher than those previously reported, yet comparable to those reported by Petropoulos et al. [43].

The differential effects of fertilizers on sugars compared with proteins and fats in garlic are attributable to distinct regulatory mechanisms of carbohydrate and protein metabolism. Garlic bulbs primarily function as storage organs and accumulate carbohydrates, particularly sugars, which are susceptible to changes depending on nutrient availability. In contrast, protein content and dry matter are more stable parameters and are less sensitive to variations induced by fertilization. Differences in nitrogen availability and form can alter carbon and nitrogen balance, leading to greater effects on sugar and sucrose accumulation than on protein or fat content.

### 2.3. Pearson’s Correlation Analysis

The correlation analysis revealed several significant (*p* < 0.05) relationships among nutritional parameters, bioactive compounds, and antioxidant activity in garlic (Table 3). Dry weight (DW) showed moderate to strong, statistically significant negative correlations with protein, sucrose, total phenolic content (TPC), and all three antioxidant assays (DPPH, FRAP, and ABTS). This indicates that garlic bulbs with higher DW tended to exhibit lower concentrations of bioactive compounds and reduced antioxidant capacity, which may suggest a dilution effect associated with increased dry matter accumulation.

Protein content was very strongly positively correlated with TPC (r = 0.82; *p* < 0.05) and showed strong positive correlations with total sugar, sucrose, TF, and antioxidant activity measured by DPPH, FRAP, and ABTS. These relationships suggest that protein-rich garlic bulbs are generally characterized by enhanced accumulation of phenolic compounds and higher antioxidant potential, reflecting coordinated metabolic regulation between nitrogen metabolism and the biosynthesis of secondary metabolites (phenolics and flavonoids). Total fat exhibited moderate, statistically significant positive correlations with total sugar and sucrose, while showing a negative correlation with TF (r = −0.51). This indicates that garlic bulbs with higher lipid content tended to accumulate more soluble carbohydrates but lower flavonoid concentrations.

Total sugar was very strongly positively correlated with sucrose and TPC (r = 0.95 and 0.80, respectively), and showed moderate to strong positive correlations with antioxidant activity, particularly ABTS, FRAP, and DPPH (Table 3). Similarly, sucrose content was strongly to very strongly positively correlated with TPC and all antioxidant assays, indicating that higher carbohydrate accumulation, particularly sucrose, is closely associated with increased phenolic content and enhanced antioxidant capacity.

Total phenolic content (TPC) exhibited strong positive correlations with TF and very strong correlations with all antioxidant assays, confirming the central role of phenolic compounds in shaping the antioxidant potential of garlic. Similar positive associations between phenolic compounds and antioxidant activity have also been reported in garlic and related *Allium* species [44,45]. Total flavonoid content (TF) showed moderate positive associations with DPPH, FRAP, and ABTS, further supporting its relevant contribution to overall antioxidant activity.

Among the antioxidant assays, DPPH showed strong positive correlations with both FRAP and ABTS, while FRAP exhibited a very strong positive correlation with ABTS (r = 0.90). These strong inter-assay correlations indicate a high level of consistency among the applied analytical methods and confirm that they reflect a common antioxidant response pattern in garlic bulbs.

### 2.4. Principal Component Analysis

**Figure 1:**
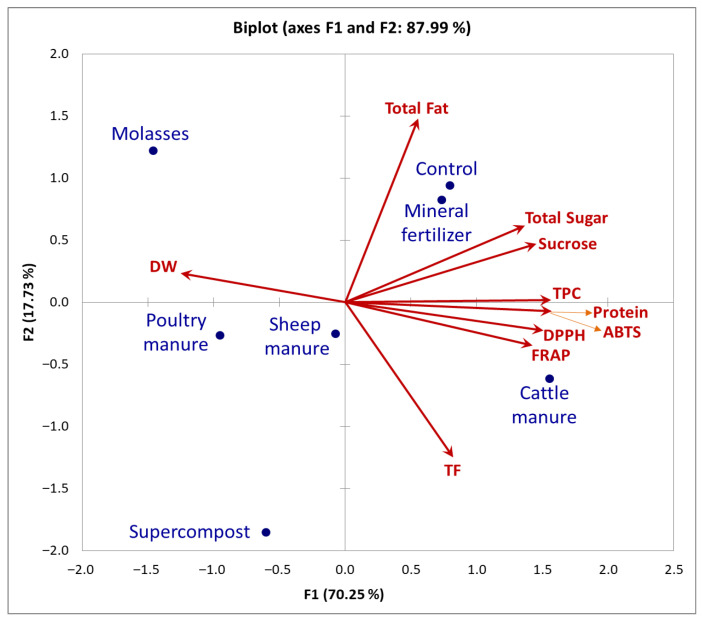
Biplot of principal components 1 and 2 (PCA dimensions F1 and F2) for nutritional parameters (DW—dry weight, protein, total fat, total sugar, sucrose), bioactive compounds (TPC—total phenolic content; TF—total flavonoid content), and antioxidant activity (DPPH, FRAP, ABTS). The biplot represents both variable loadings and treatment scores, allowing visualization of relationships among traits and fertilization treatments. Control refers to the treatment without any fertilizer application (0 t/ha).

The PCA results indicated that the first three principal components (PCA dimensions F1, F2, and F3) together accounted for 94.49% of the total variance in the nutritional composition, bioactive compounds, and antioxidant activity of garlic. The first principal component (F1) explained 70.25% of the variance, followed by 17.73% for F2 and 6.50% for F3. According to the Kaiser criterion (eigenvalue > 1), F1 and F2 were identified as the most informative dimensions, jointly explaining 87.99% of the total variability (Figure 1), while inclusion of F3 further improved the overall description of the dataset structure.

The contribution of individual variables (nutritional parameters, bioactive compounds, and antioxidant activity) to the PCA dimensions showed that dimension F1 was predominantly defined by DW, protein, total sugar, sucrose, TPC, DPPH, FRAP, and ABTS, which together contributed 94.58% to its variability. The greatest contribution within F1 was recorded for ABTS (13.55%), followed by TPC, protein, DPPH, sucrose, FRAP, total sugar, and DW. These findings indicate that F1 primarily represents a dominant quality-related gradient, reflecting the coordinated accumulation of sugars, phenolic compounds, and antioxidant capacity, positioned oppositely to DW. This is consistent with the Pearson correlation analysis, confirming an inverse relationship between DW and biochemical quality traits. The second PCA dimension (F2) was mainly influenced by total fat and TF, which together accounted for 81.72% of the variability explained by this axis. Other traits contributed minimally to F2, indicating that their major variability had already been captured by F1. Therefore, F2 predominantly represents variation associated with the lipid fraction and flavonoid content of garlic bulbs. The third PCA dimension (F3), although below the eigenvalue > 1 threshold, was primarily associated with DW and was strongly driven by the sheep manure treatment, indicating specific variability patterns not emphasized in the first two components.

The PCA of fertilization treatments, as depicted in the F1 × F2 biplot (Figure 1), provides a comprehensive overview of the relationships among nutritional parameters, bioactive compounds, and antioxidant traits, as well as the differentiation of fertilizer treatments based on these characteristics. In the PCA biplot, which combines variable loadings and treatment scores, each trait is represented by a vector originating from the origin. The correlation between any two traits is approximated by the cosine of the angle between their vectors, where acute angles indicate positive relationships, obtuse angles indicate negative associations, and right angles indicate little or no correlation [46].

In the F1 dimension, the relationships between traits are clearly evident from vector orientations (Figure 1). FRAP, DPPH, ABTS, protein, TPC, sucrose, and total sugar were closely grouped, with narrow angles between their vectors, indicating strong positive associations among these traits. In contrast, DW was oriented in the opposite direction, confirming its strong negative association with these antioxidant- and phenolic-related parameters, which corroborates the Pearson’s correlation results (Table 3). Along the second PCA dimension (F2), total fat and TF contributed most to variability, confirming that this axis primarily reflects variation associated with the lipid and flavonoid components of garlic quality.

Considering the contribution of the observations (fertilization treatments), cattle manure was the dominant contributor along PCA dimension F1 (34.55%) and was positioned in the direction of higher antioxidant activity (DPPH, FRAP, ABTS), as well as higher protein, TPC, sucrose, and total sugar, but opposite to DW. Molasses and poultry manure also contributed markedly to F1 (30.43% and 12.95%, respectively), with molasses simultaneously showing relevance to F2, reflecting its dual influence on DW and biochemical traits. In contrast, supercompost was positioned predominantly along F2 (48.94%), being associated mainly with higher TF, while the control and mineral fertilizer treatments were similarly aligned with traits such as total fat, total sugar, sucrose, and TPC. Sheep manure was specifically distinguished along F3 through its relationship with DW.

Overall, the PCA clearly demonstrated that fertilization regimes differentially influenced garlic’s nutritional composition, bioactive compound accumulation, and antioxidant potential. PCA dimension F1 described the dominant contrast between DW and antioxidant- and phenolic-related traits, F2 primarily differentiated treatments according to total fat and TF, while F3 reflected specific variability particularly associated with DW under the sheep manure treatment.

### 2.5. Cluster Analysis

**Figure 2:**
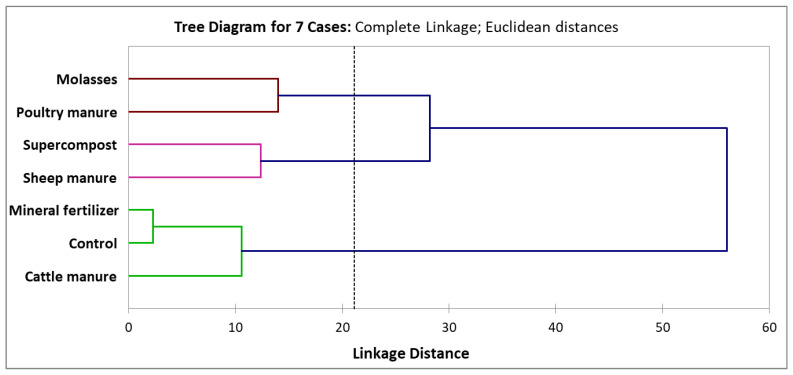
Dendrogram from agglomerative hierarchical clustering (complete linkage) of fertilization treatments based on garlic nutritional composition, bioactive compounds, and antioxidant activity. The clusters are color-coded as follows: brown (Cluster 1), pink (Cluster 2), and green (Cluster 3). Control refers to treatment without any fertilizer application (0 t/ha).

Grouping of fertilization treatments based on the nutritional composition, bioactive compounds, and antioxidant activity of garlic was carried out using agglomerative hierarchical clustering with the complete linkage method. The dendrogram obtained from the cluster analysis showed that the treatments were initially divided into two main clusters (Figure 2): one comprising molasses, poultry manure, supercompost, and sheep manure, and the other including mineral fertilizer, control, and cattle manure.

All fertilization regimes were subsequently separated into three distinct clusters. The first cluster included molasses and poultry manure, which were characterized by the lowest antioxidant capacity (DPPH, FRAP, ABTS), together with the lowest phenolic-related traits (TPC and TF) and protein content, as well as comparatively reduced total sugar and sucrose contents. These treatments were therefore associated with a weaker expression of biochemical quality traits. The second cluster grouped supercompost and sheep manure, which exhibited the lowest total fat, total sugar, and sucrose contents, and intermediate antioxidant performance, accompanied by moderate levels of bioactive compounds (TPC and TF). This cluster thus represented an intermediate biochemical profile between the lowest- and highest-performing fertilization strategies. The third cluster comprised mineral fertilizer, control, and cattle manure, notable for the highest protein, total fat, total sugar and sucrose contents, as well as antioxidant activity (DPPH, FRAP, ABTS), and the greatest TPC and TF values, clearly indicating that these fertilization strategies were associated with the most pronounced nutritional composition and biochemical quality of garlic.

The obtained clustering pattern largely corroborated the results of the PCA. Both analyses clearly positioned the control, mineral fertilizer, and cattle manure treatments in close association with antioxidant- and phenolic-related traits, while sheep manure and supercompost were similarly differentiated from these treatments, reflecting their specific biochemical profile. Molasses and poultry manure were located away from the group of treatments associated with the strongest antioxidant response, indicating their comparatively weaker biochemical performance. Such consistency between PCA and hierarchical clustering confirms the robustness of the multivariate structure of the dataset and the clear differentiation of fertilization treatments according to their influence on garlic nutritional composition, phenolic accumulation, and antioxidant potential. Comparable clustering patterns based on biochemical quality traits were previously observed in garlic and celery under different fertilization regimes [20,47].

### 2.6. Content of Macro- and Microelements in Garlic

The average N content in garlic was 2.73%, ranging from 2.64 (molasses) to 2.81% (sheep manure) (Table 4) exceeding those reported by Yadav et al. [48]. Fertilization did not result in significant differences in garlic N content compared with the control treatment. However, significantly higher N concentrations were observed in the cattle manure and sheep manure treatments compared with supercompost and molasses treatments. Nitrogen accumulation in plant tissues is strongly influenced by soil N availability, which is largely determined by the type and maturity of organic amendments [49].

The P content observed in this study was higher than previously reported [38,48], but slightly lower than the values reported by Ismail et al. [50]. Fertilization increased P content in the mineral fertilizer, cattle manure, sheep manure, and supercompost treatments compared with the control, although these differences were not statistically significant, while a decrease was observed in the molasses treatment. This effect may be attributed to the higher solubility and faster nutrient release associated with mineral fertilizers [49]. Phosphorus was not applied in the molasses treatment (Table 4), while the presence of polysaccharides in molasses may have influenced microbial activity involved in the mineralization of organic phosphorus compounds [51]. Compared with the mineral fertilizer treatment, all treatments exhibited lower P content, except for sheep manure, which revealed similar values (Table 4). Variations in P content as influenced by fertilization and production system have also been reported by Bajpai and Punia [40], who found higher P concentrations in organic production systems compared with inorganic and conventional systems.

Fertilization did not significantly affect K content in garlic, except in the molasses treatment, which showed a reduction in K content. Potassium concentrations ranged from 1.31 (molasses) to 1.36% (sheep manure) (Table 4), substantially higher than previously reported values [39,48,50]. All organic fertilizer treatments, except molasses, resulted in higher K content compared with the mineral fertilizer treatment. Increased K_2_O availability has been linked to enhanced soil biological activity, which can be promoted through the application of organic fertilizers [52,53]. Potassium content in garlic is also influenced by K fertilization, as shown by Ismail et al. [50], who reported increased K accumulation with higher K_2_O application rates. Additionally, K content varies among genotypes, as reported by Petropoulos et al. [43], who recorded higher K concentrations in 14 Greek garlic landraces.

Calcium content was higher than the previously reported values [39,48,50], but significantly lower than those reported by Petropoulos et al. [43]. Similar Ca levels were observed in the control, mineral fertilizer, and molasses treatments, whereas lower, non-significant differences occurred in other treatments (Table 4). Variations in Ca accumulation may be related to antagonistic interactions between Ca and K influenced by the K_2_O content of applied fertilizers [54,55]. Higher Ca contents in garlic under organic production systems compared with inorganic and conventional systems have been previously reported [40], with similar trends observed for garlic, leek, red onion, and Welsh onion [18].

The magnesium content in garlic observed in this study was higher than previously reported values [18,39,40], but lower than those reported by Petropoulos et al. [43]. Compared with the control, fertilization with organic fertilizers significantly reduced Mg content in garlic, except in the cattle manure treatment, where the reduction was not statistically significant. The application of mineral fertilizer did not affect Mg content, as values were similar to those of the control (Table 4). In contrast, Bajpai and Punia [40] reported higher Mg content in organically produced garlic compared with inorganic and conventional systems, and similar trends were observed in garlic, leek, red onion, and Welsh onion [18].

Fertilization with cattle manure and sheep manure significantly increased S content in garlic bulbs, whereas all remaining fertilization treatments resulted in S concentrations that did not differ significantly from the control (Table 4). The sulfur content observed in this study was significantly higher than the values reported by Yadav et al. [48], while Nguyen et al. [56] reported similar or significantly higher sulfur contents depending on genotype. These authors further indicated that sulfur content in garlic dry matter directly affects the accumulation of key sulfur-containing compounds, such as alliin, allicin, and thiosulfinates, with higher sulfur levels in dry matter being associated with increased concentrations of these bioactive compounds.

The small differences observed among treatments in the contents of N, P, K, Ca, Mg, and S in garlic bulbs are consistent with the limited effects of treatments on dry weight and protein content. Since plant growth and protein synthesis were only slightly affected, the uptake and accumulation of mineral nutrients also remained relatively stable across treatments. This suggests that garlic maintains a relatively constant elemental composition under different fertilization regimes. Although sulfur is essential for the formation of bioactive compounds in garlic, its total concentration in the bulbs did not vary markedly among treatments. This indicates that differences in antioxidant activity and bioactive properties are more likely related to metabolic and enzymatic regulation rather than to changes in total sulfur content. In contrast, carbohydrate-related traits (sugar and sucrose) were more responsive to fertilization treatments, suggesting that the observed differences in garlic quality are mainly driven by metabolic adjustments rather than by major changes in mineral nutrient composition.

Fertilizer application reduced B content in garlic, with a statistically significant decrease observed for all organic fertilizer treatments, while the reduction observed in the mineral fertilizer treatment was not statistically significant. The greatest reduction, 23.0%, was recorded in the molasses treatment (Table 5). There is limited information in the scientific literature regarding the effects of organic and mineral fertilizers on B content in garlic. El-Bahloul et al. [57] reported that increasing K_2_O rates and availability enhanced B accumulation, which contrasts with the present findings, as the molasses treatment received the highest K_2_O input but exhibited the lowest B content. The same authors also reported substantially higher B concentrations in garlic compared with the values recorded in this study.

The application of sheep manure significantly increased Cu content in garlic compared with the control, whereas the application of supercompost and molasses resulted in a significant reduction in Cu content (Table 5). No significant differences in Cu content were observed among the mineral fertilizer, cattle manure, and poultry manure treatments. These results are partially consistent with findings previously reported in [18,40], who observed higher Cu concentrations in organically produced garlic compared with inorganic and conventional production systems.

The average Fe content ranged from 48.47 to 71.84 mg/kg (Table 5). Similar values were reported by Czech et al. [18], whereas lower Fe concentrations were reported in previous studies [39,40,50]. Fertilization significantly reduced Fe content in garlic. Mineral fertilizer application decreased Fe content by 20.3%, while the application of supercompost and molasses resulted in reductions of 27.6% and 17.0%, respectively. Compared with the mineral fertilizer treatment, higher Fe content was recorded in the cattle manure and molasses treatments, whereas lower Fe concentrations were observed in the sheep manure, poultry manure, and supercompost treatments. These findings are partially consistent with those reported by Bajpai and Punia [40] and Czech et al. [18], who observed higher Fe concentrations in garlic, red onion, leek, and Welsh onion grown under organic production systems compared with inorganic and conventional systems. Differences in iron content between organically and inorganically grown vegetables may be explained by the predominance of poorly available iron forms in soils under inorganic management, whereas higher microbial activity in organically managed soils promotes the production of organic compounds, such as citrate and lactate, which chelate Fe and enhance its availability to plant roots [58].

Fertilization did not significantly affect Mn content in garlic, except in the poultry manure treatment, where a significant reduction of 13.1% relative to the control was observed (Table 5). The highest Mn concentration was recorded in the mineral fertilizer treatment and was higher than that measured in the organic fertilizer treatments, among which no significant differences were observed. These results are not consistent with previous reports [18,40]. Mn content depends on plant species [21] as well as genotype [32], and the Mn concentrations recorded in this study exceeded those reported in previous studies [18,39,40].

The highest Na content was observed in the control treatment; whereas both mineral and organic fertilization resulted in significantly lower Na concentrations in garlic bulbs (Table 5). This is consistent with findings reported for *Allium* species, where improved nutrient supply reduced Na accumulation through competitive ion uptake and dilution effects [35]. Sajid et al. [39] and Eissa et al. [38] reported lower Na concentrations than those observed in the present study, while Petropoulos et al. [43] reported significant genotype-dependent differences in sodium content.

The application of sheep manure increased Zn content by 116.4% relative to the control, while the application of the remaining fertilizers resulted in changes that were not statistically significant (Table 5). No significant differences were observed between the mineral fertilizer treatment and the other organic fertilizer treatments. These findings contrast with those reported by Bajpai and Punia [40] and Czech et al. [18]. Although several studies have reported Zn concentrations in garlic, the values are generally lower than those obtained in the present study [18,38,39,40], which may be attributed to genotype-related differences [43]. Copper and Zn are common constituents of animal feed supplements, and differences in their concentrations among treatments receiving animal-based organic fertilizers may reflect variations in animal diets, providing additional sources for soil and plant uptake [59,60].

## 3. Materials and Methods

### 3.1. Experimental Design and Plant Material

The randomized complete block design experiment was conducted under open-field conditions at the Institute of Field and Vegetable Crops (45°06′ N; 19°30′ E; 80 m a.s.l.), in Novi Sad, Vojvodina Province, Republic of Serbia. It was set in 3 replications. The garlic variety “Ranko”, developed by the Institute of Field and Vegetable Crops in Novi Sad, was used in the experiment. It is an autumn variety intended for the production of both green garlic and bulbs. It is characterized by compact bulbs containing 8 to 10 cloves arranged in a circular pattern. The experimental plot size was 7 m^2^, within which the study was conducted on plots of 3.5 m^2^. Manual planting of garlic cloves was carried out on 26 October 2023. The cloves were planted at a spacing of 35 × 10 cm. Each experimental plot contained 200 plants, while 100 plants were included in the accounting plot. The preceding crop for the garlic was celeriac, which had been fertilized with mineral fertilizers, organic fertilizers of animal origin, and by-products from the food industry. The garlic itself was not directly fertilized; instead, it utilized the residual effects of the fertilizers applied five months earlier, in May 2023, prior to celeriac planting. Organic fertilizers require a longer period to decompose in soil and to convert nutrients from organic forms into mineral forms that are available for plant uptake. Organic fertilizers exhibit a prolonged residual effect lasting several years, with the most pronounced impact occurring during the first year after application. This application timing was chosen to ensure a more balanced nutrient supply during garlic growth. Harvesting was conducted on 2 July 2024, when approximately 50% of the aboveground garlic foliage had lodged. For the determination of the analyzed parameters, all plants were used to create a composite sample for further laboratory analyses.

### 3.2. Agrochemical Properties at the Experimental Site

Agrochemical analyses of both the soil and the organic fertilizers were performed before fertilizer application and the results are presented in Table 6 and Table 7.

Agrochemical analysis of the soil (Table 6) showed that the soil was slightly alkaline, according to Thun’s classification. In terms of calcium carbonate content, the soil was categorized as a carbonate soil [61]. Based on the Scheffer–Schachtschabel classification, it was identified as a low-humus soil. According to the total nitrogen content, the soil was classified as poorly to moderately supplied with nitrogen [62]. Phosphorus and potassium levels were very high, and readily available potassium content was high [63]. These soil characteristics provide important context for assessing the effects of different fertilization treatments.

Supercompost is a sludge that is produced as a byproduct in a wastewater treatment plant at the “Carlsberg” brewery in Čelarevo, Republic of Serbia. It is formed through the fermentation of organic material of plant origin, has a gray-brown color, and a mildly alkaline reaction. The heavy metal content is below the maximum allowed concentration, making it suitable for agricultural use.

Molasses is the syrup that remains after the crystallization of sugar from sugar beet. The syrup is a thick, viscous, dark-colored product. It is suitable for fertilizing the soil because it has a rich content of mineral substances, which may improve plant growth, and the sugar residues stimulate the activity of microorganisms.

The chemical composition of manures and organic by-products can fluctuate significantly based on animal diets, processing methods, and seasonal factors. To mitigate this variability and ensure the reliability of test results, we utilized single, thoroughly homogenized batches of each fertilizer. All materials were chemically characterized prior to application, and application rates were precisely calculated based on these results. This approach ensured that the observed differences in garlic composition were attributable to the fertilizer type rather than batch-to-batch inconsistencies. Based on these analyses, the amounts of organic fertilizers were determined to ensure a uniform nitrogen input of 170 kg N/ha across all treatments (except the control, which received no application). This nitrogen rate represents the maximum allowable amount in organic production. According to the Nitrate Directive [64], the maximum permissible annual nitrogen application in nitrate-sensitive zones is 170 kg N/ha. The applied fertilizer rates for the preceding crop are presented in Table 8.

### 3.3. Weather Conditions

**Figure 3:**
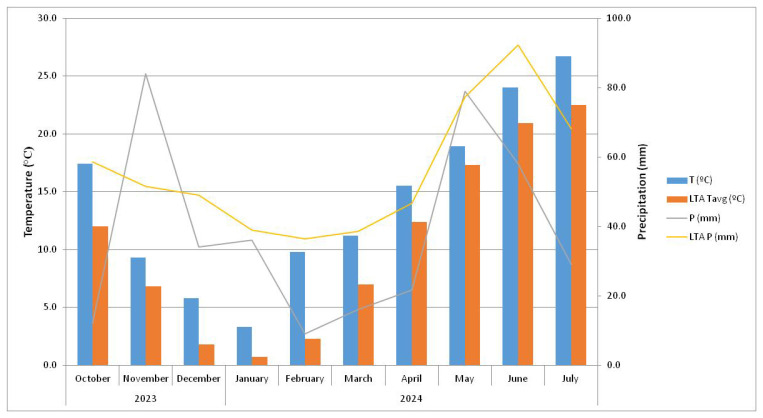
Meteorological conditions during the experiment (from October 2023 to July 2024). The blue bars represent average monthly temperature, while the orange bars indicate the long-term average monthly temperature (1991–2020). The gray line represents total monthly precipitation, while the yellow line represents the long-term average precipitation sum (1991–2020).

During the experiment, the highest average monthly temperature was recorded in July (26.7 °C), which was 3.7 °C higher than the long-term average (1991–2020) (Figure 3). The lowest temperature was measured in January (3.3 °C), which was 2.6 °C higher than the long-term average. In terms of precipitation, the highest amount was recorded in November (84.0 mm), which was 28.5 mm more than the long-term average. The lowest amount of precipitation was in February (9.0 mm), which was 27.4 mm less than the long-term average. Overall, the observed climatic conditions, characterized by higher-than-average winter temperatures and sufficient precipitation during the growing season, were generally favorable for garlic production.

### 3.4. Measurements and Analytical Determination

The soil pH was determined in a soil-to-water suspension (1:2.5) using a Metrel MA 3657 pH meter (Mettler Toledo, Horjul, Slovenia), following the ISO 10390 standard [65]. Soil calcium carbonate (CaCO_3_) content was quantified volumetrically with a Scheibler calcimeter (Gabbrielli, Mie, Japan). Soil humus content was determined according to the Tyurin method [62]. Total nitrogen (N) content in the soil was analyzed with a CHNS analyzer (Elementar Vario EL, GmbH, Hanau, Germany). Plant-available phosphorus (P) and potassium (K) were extracted using an AL solution (0.1 M ammonium lactate and 0.4 M acetic acid, pH 3.75) at a soil-to-solution ratio of 1:20 (*w*/*v*) [63].

The dry matter content of the organic fertilizers was determined gravimetrically by drying at 100–105 °C. Nitrogen and organic carbon in the organic fertilizers were measured using a CHNS analyzer (Elementar Vario EL, GmbH, Hanau, Germany). Potassium concentrations were determined after wet digestion with a HNO_3_: HClO_4_ mixture (4:1, *v*/*v*) using a flame atomic absorption spectrometer (Shimadzu 6300, Kyoto, Japan), whereas phosphorus concentrations were measured colorimetrically with a Jenway 6105 spectrophotometer (Jenway Ltd., Dunmow, UK). The organic matter was determined using the loss on ignition method (EN 13040:2007) [66].

The dry weight (DW) content of all garlic bulb samples was determined gravimetrically by drying the material at 105 ± 0.5 °C until a constant mass was achieved [67]. All measurements were performed in triplicate, and the results were reported as the mean dry weight in percentage.

Prior to extraction, the samples of garlic bulbs were homogenized using a basic mill (A11BS000, IKA, Staufen, Germany). The garlic samples were subsequently transferred into 50 mL Erlenmeyer flasks, to which 25 mL of methanol was added as the extraction solvent. The flasks were sealed with aluminum foil and placed on a shaker (UNIMAX 1010, Heidolph, Schwabach, Germany), where they were agitated at 100 rpm for 24 h in the dark at room temperature. Following extraction, the mixtures were quantitatively transferred into 50 mL volumetric flasks and brought to volume with methanol. The solutions were then filtered through qualitative filter paper to obtain clear extracts. All extracts were stored at 4 °C until further analysis. These prepared extracts were subsequently used for the determination of total phenolic content (TPC), total flavonoid content (TF), and antioxidant activity.

The total phenolic content (TPC) in all garlic samples was quantified spectrophotometrically using the Folin–Ciocalteu method [68], with gallic acid serving as the calibration standard. Results were expressed as gallic acid equivalents (mg GAE/100 g DW).

The total flavonoid content (TF) was determined spectrophotometrically by the aluminum chloride colorimetric assay [69]. The results were expressed as catechin equivalents (mg CE/100 g DW).

The free radical-scavenging activity of all garlic samples was assessed using the spectrophotometric procedure described by Espín et al. [70]. The prepared garlic extract was mixed with 96% methanol and 90 μM 2,2-diphenyl-1-picrylhydrazyl (DPPH). After incubation for 60 min at room temperature, the absorbance was recorded at 517 nm.

The Ferric Reducing Antioxidant Power (FRAP) assay was employed to assess the ability of the garlic extracts to reduce trivalent ferric ions (Fe^3+^) [71]. The freshly prepared FRAP reagent consisted of 10 mM TPTZ (2,4,6-tris(2-pyridyl)-s-triazine) in 40 mM HCl, a 20 mM aqueous solution of iron(III)-chloride (FeCl_3_), and a 300 mM acetate buffer adjusted to pH 3.6. The absorbance of the reaction mixture was subsequently measured at 593 nm after 10 min incubation at 37 °C in the dark [71].

The antioxidant activity based on the 2,2′-azino-bis(3-ethylbenzothiazoline-6-sulfonic acid) diammonium salt (ABTS) assay was performed according to a modified procedure described by Re et al. [72] The ABTS reagent was prepared by reacting a 7 mM aqueous ABTS solution with a 2.45 mM potassium persulfate solution and allowing the mixture to stand in the dark at room temperature for 16 h. For the assay, 2.9 mL of the ABTS reagent was mixed with 0.1 mL of the diluted extracts and incubated for 300 min at room temperature in the dark. Following incubation, absorbance was measured at 734 nm [72]. Depending on the antioxidant assay, the results for DPPH (mg Trolox (TC)/100 g DM), FRAP (mg Fe^2+^/100 g DM), and ABTS (mg TC/100 g DM) are interpreted such that higher values correspond to greater antioxidant activity.

The nutritional composition of the plant samples was analyzed by determining fat, protein, total sugars, and minerals. The procedure for determining the fat content in garlic samples was carried out according to the Soxhlet extraction method [73], using diethyl ether as a solvent. Protein content in the garlic samples was determined by the Kjeldahl method described in the study by Marcó et al. [74]. The content of total sugars and sucrose in garlic samples was analyzed according to the Luff–Schoorl method [63]. All measurements of fat, protein, and total sugar content were repeated in three times, and the results are reported as mean percentage values. The content of macro and microelements in plant material was determined by the inductively coupled plasma method (ICP method) [75].

### 3.5. Statistical Analysis

The research results were analyzed using the analysis of variance (ANOVA) method for a one-factor experiment, using the statistical software “Statistica 14”, and the significance of differences between the means of the treatments was tested using Tukey’s HSD test at the level of significance α = 0.05. Lowercase letters mark significance in the difference among fertilizing variants. Correlation analysis among the analyzed parameters was calculated using Pearson’s correlation at the significance level α = 0.05. Pearson’s correlation analysis was applied following standard statistical approaches [76] and similar methodologies used in garlic and *Allium* studies [44,45]. To determine the relationships between the various parameters and to identify the set of parameters that explain the maximum variability among the fertilizing treatments, the multivariate data analysis method PCA (Principal Component Analysis) was used. Grouping of fertilizing treatments based on nutritional value, bioactive compounds, and antioxidant activity was carried out using Cluster analysis (hierarchical clustering), employing the complete linkage agglomeration method. Hierarchical cluster analysis (HCA) was conducted according to established methodological frameworks [77] and recent agricultural applications combining PCA and HCA approaches [20,45,47]. Statistical analyses were performed using the Statistica software package, version 14 (TIBCO Software Inc., Palo Alto, CA, USA) and GenStat for Windows 12th Ed. (VSN International, Hemel Hempstead, UK).

## 4. Conclusions

The results demonstrate that fertilization regimes play a crucial role in determining garlic quality. Animal-based organic fertilizers, particularly cattle manure, represent a sustainable alternative to mineral fertilization, enabling the production of garlic with improved nutritional and mineral profiles, elevated levels of bioactive compounds, and enhanced antioxidant activity. By-products from the food processing industry, supercompost and sugar beet molasses, increased garlic dry weight; however, they resulted in reduced protein, total fat, total sugars, sucrose, total phenolic and flavonoid contents, as well as antioxidant activity, compared with the control. Their application markedly altered the mineral profile, leading to decreased content of most analyzed elements, with the exception of phosphorus under supercompost treatment and calcium under molasses treatment. Multivariate analyses demonstrated that fertilization regimes significantly affected garlic quality, with cattle manure, mineral fertilizer, and control promoting higher bioactive compounds, antioxidant activity, and nutritional composition. FRAP, DPPH, ABTS, protein, TPC, sucrose, and total sugar clustered closely, with small angles between their vectors, indicating strong positive relationships among these traits. Conversely, DW was oriented in the opposite direction, demonstrating a pronounced negative association with antioxidant- and phenolic-related parameters, consistent with the Pearson correlation results. These findings indicate that organic fertilizers cannot be considered a homogeneous group with respect to food quality outcomes. Therefore, careful selection of fertilizer type is essential to ensure the production of garlic with high nutritional and functional value for human consumption.

## Figures and Tables

**Table 1:** Content of bioactive compounds and antioxidant activity of garlic.

Treatments		TPC	TF	DPPH	FRAP	ABTS
Control		150.99 ± 2.47 ^a^	27.44 ± 0.85 ^b^	0.14 ± 0.01 ^a^	0.13 ± 0.00 ^b^	0.58 ± 0.01 ^a^
Mineral fertilizer		152.29 ± 1.14 ^a^	28.24 ± 2.33 ^b^	0.13 ± 0.01 ^a^	0.11 ± 0.00 ^c^	0.54 ± 0.00 ^b^
Cattle manure		155.57 ± 0.65 ^a^	35.85 ± 2.44 ^a^	0.15 ± 0.03 ^a^	0.16 ± 0.00 ^a^	0.61 ± 0.01 ^a^
Sheep manure		127.18 ± 0.29 ^b^	24.67 ± 0.93 ^bc^	0.13 ± 0.01 ^a^	0.13 ± 0.00 ^b^	0.52 ± 0.02 ^bc^
Poultry manure		115.22 ± 2.46 ^c^	27.64 ± 3.65 ^b^	0.09 ± 0.00 ^b^	0.10 ± 0.00 ^de^	0.45 ± 0.01 ^d^
Supercompost		126.61 ± 5.98 ^b^	36.21 ± 2.57 ^a^	0.12 ± 0.00 ^ab^	0.11 ± 0.00 ^c^	0.50 ± 0.01 ^c^
Molasses		103.92 ± 1.02 ^d^	20.01 ± 1.33 ^c^	0.08 ± 0.00 ^b^	0.09 ± 0.00 ^e^	0.44 ± 0.00 ^d^
Average		133.11 ± 19.27	28.58 ± 5.81	0.12 ± 0.03	0.12 ± 0.02	0.52 ± 0.06
CV (%)		14.48	20.34	23.30	17.53	11.64
ANOVA (*n* = 21)	R^2^	0.99	0.90	0.80	0.98	0.98
	F	168.29	20.45	9.35	136.47	103.60
	*p*	<0.001	<0.001	0.000	<0.001	<0.001

Control refers the treatment without any fertilizer application (0 t/ha), TPC—Total phenolic content (mg/100 g DW), TF—Total flavonoid content (mg/100 g DW), DPPH—2,2-diphenyl-1-picrylhydrazyl radical scavenging assay for antioxidant activity (mg/g DW), FRAP—The Ferric Reducing Antioxidant Power (mg Fe^2+^/g DW), ABTS—2,2′-azino-bis(3-ethylbenzothiazoline-6-sulfonic acid) radical cation decolorization assay for antioxidant activity (mg/g DW), R^2^—coefficient of determination, F—variance ratio (F-test), *p*—probability value corresponding to a variance ratio. Mean value ± standard deviation (SD). Values marked with the same letter in a column are not significantly different at 5% (Tukey’s HSD test).

**Table 2:** Nutritional value of garlic.

Treatments		DW (%)	Protein (%)	Total Fat (%)	Total Sugar (%)	Sucrose (%)
Control		31.19 ± 0.01 ^c^	5.49 ± 0.23 ^bc^	0.24 ± 0.01 ^a^	17.49 ± 0.09 ^b^	12.58 ± 0.03 ^b^
Mineral fertilizer		30.65 ± 0.01 ^e^	5.65 ± 0.22 ^ab^	0.24 ± 0.02 ^a^	17.58 ± 0.34 ^b^	11.01 ± 0.22 ^c^
Cattle manure		30.97 ± 0.13 ^d^	5.77 ± 0.06 ^a^	0.15 ± 0.02 ^c^	20.62 ± 0.16 ^a^	15.71 ± 0.27 ^a^
Sheep manure		30.58 ± 0.07 ^e^	5.39 ± 0.14 ^b–d^	0.16 ± 0.01 ^c^	8.99 ± 0.14 ^d^	6.32 ± 0.25 ^d^
Poultry manure		31.81 ± 0.00 ^b^	5.27 ± 0.19 ^cd^	0.13 ± 0.02 ^c^	9.12 ± 0.21 ^d^	6.30 ± 0.03 ^d^
Supercompost		31.89 ± 0.06 ^b^	5.32 ± 0.03 ^cd^	0.08 ± 0.02 ^d^	7.83 ± 0.09 ^e^	2.29 ± 0.05 ^f^
Molasses		33.23 ± 0.04 ^a^	5.14 ± 0.07 ^d^	0.20 ± 0.03 ^b^	11.28 ± 0.12 ^c^	4.45 ± 0.22 ^e^
Average		31.47 ± 0.88	5.43 ± 0.24	0.17 ± 0.06	13.27 ± 4.89	8.38 ± 4.58
CV (%)		2.81	4.50	34.32	36.89	54.60
ANOVA (*n* = 21)	R^2^	1.00	0.72	0.94	1.00	1.00
	F	669.06	6.06	36.73	2404.28	2050.54
	*p*	<0.001	0.003	<0.001	<0.001	<0.001

Control refers to treatment without any fertilizer application (0 t/ha), DW—Dry weight, R^2^—coefficient of determination, F—variance ratio (F-test), *p*—probability value corresponding to a variance ratio. Mean value ± standard deviation (SD). Values marked with the same letter in a column are not significantly different at 5% (Tukey’s HSD test).

**Table 3:** Pearson’s correlation coefficients (r) among nutritional parameters, bioactive compounds, and antioxidant activity traits in garlic *.

Variables	Protein	Total Fat	Total Sugar	Sucrose	TPC	TF	DPPH	FRAP	ABTS
DW	***−0.64***	−0.16	−0.41	***−0.57***	***−0.77***	−0.36	***−0.78***	***−0.66***	***−0.71***
Protein	1	0.29	***0.71***	***0.74***	***0.82***	***0.51***	***0.57***	***0.73***	***0.73***
Total Fat	-	1	***0.60***	***0.52***	0.37	***−0.51***	0.16	0.05	0.28
Total Sugar	-	-	1	***0.95***	***0.82***	0.23	***0.59***	***0.66***	***0.80***
Sucrose	-	-	-	1	***0.84***	0.25	***0.65***	***0.78***	***0.85***
TPC	-	-	-	-	1	***0.53***	***0.83***	***0.77***	***0.92***
TF	-	-	-	-	-	1	***0.49***	***0.53***	***0.51***
DPPH	-	-	-	-	-	-	1	***0.72***	***0.87***
FRAP	-	-	-	-	-	-	-	1	***0.90***

* Bolded values in italics are different from 0 with a significance level α = 0.05. Sample size: *n* = 21. DW—dry weight, TPC—Total phenolic content (mg/100 g DW), TF—Total flavonoid content (mg/100 g DW), DPPH—2,2-diphenyl-1-picrylhydrazyl radical scavenging assay for antioxidant activity (mg/g DW), FRAP—The Ferric Reducing Antioxidant Power (mg Fe^2+^/g), ABTS—2,2′-azino-bis(3-ethylbenzothiazoline-6-sulfonic acid assay for antioxidant activity (mg/g DW).

**Table 4:** Content of macroelements (%).

Treatments		N	P	K	Ca	Mg	S
Control		2.704 ± 0.013 ^a–c^	0.405 ± 0.006 ^ab^	1.352 ± 0.005 ^a^	0.076 ± 0.002 ^a^	0.079 ± 0.002 ^a^	0.752 ± 0.005 ^c^
Mineral fertilizer		2.732 ± 0.024 ^a–c^	0.434 ± 0.036 ^a^	1.330 ± 0.013 ^ab^	0.083 ± 0.021 ^a^	0.079 ± 0.004 ^a^	0.767 ± 0.001 ^bc^
Cattle manure		2.804 ± 0.007 ^ab^	0.419 ± 0.002 ^a^	1.354 ± 0.001 ^a^	0.071 ± 0.000 ^a^	0.077 ± 0.001 ^ab^	0.806 ± 0.007 ^ab^
Sheep manure		2.814 ± 0.006 ^a^	0.435 ± 0.002 ^a^	1.357 ± 0.012 ^a^	0.071 ± 0.000 ^a^	0.075 ± 0.000 ^bc^	0.822 ± 0.022 ^a^
Poultry manure		2.694 ± 0.035 ^bc^	0.404 ± 0.000 ^ab^	1.338 ± 0.009 ^ab^	0.070 ± 0.001 ^a^	0.072 ± 0.000 ^c^	0.763 ± 0.015 ^bc^
Supercompost		2.678 ± 0.053 ^c^	0.410 ± 0.005 ^ab^	1.344 ± 0.001 ^a^	0.061 ± 0.001 ^a^	0.074 ± 0.001 ^bc^	0.743 ± 0.010 ^c^
Molasses		2.638 ± 0.079 ^c^	0.373 ± 0.008 ^b^	1.306 ± 0.027 ^b^	0.076 ± 0.003 ^a^	0.074 ± 0.002 ^bc^	0.736 ± 0.038 ^c^
Average		2.723 ± 0.070	0.411	1.340	0.072	0.076	0.770
CV (%)		2.573	5.679	1.478	12.629	3.822	4.425
ANOVA (*n* = 21)	R^2^	0.777	0.737	0.716	0.481	0.739	0.805
	F	8.126	6.546	5.886	2.166	6.598	9.636
	*p*	0.001	0.002	0.003	0.110	0.002	0.000

Control refers the treatment without any fertilizer application (0 t/ha), N—nitrogen, P—phosphorus, K—potassium, Ca—calcium, Mg—magnesium, S—sulphur, R^2^—coefficient of determination, F—variance ratio (F-test), *p*—probability value corresponding to a variance ratio. Mean value ± standard deviation (SD). Values marked with the same letter in a column are not significantly different at 5% (Tukey’s HSD test).

**Table 5:** Content of microelements (mg/kg).

Treatments		B	Cu	Fe	Mn	Na	Zn
Control		11.860 ± 0.057 ^a^	6.005 ± 0.455 ^c^	71.840 ± 4.270 ^a^	10.905 ± 0.015 ^a^	710.600 ± 4.100 ^a^	27.060 ± 2.710 ^b^
Mineral fertilizer		11.450 ± 0.340 ^a^	6.450 ± 0.170 ^a–c^	57.250 ± 1.960 ^b^	10.955 ± 1.205 ^a^	630.100 ± 5.200 ^c^	31.050 ± 2.820 ^ab^
Cattle manure		10.420 ± 0.020 ^b^	6.910 ± 0.250 ^ab^	59.245 ± 0.655 ^b^	10.580 ± 0.180 ^ab^	670.500 ± 17.30 ^b^	30.030 ± 3.230 ^ab^
Sheep manure		10.170 ± 0.030	7.290 ± 0.990 ^a^	54.595 ± 1.015 ^bc^	10.140 ± 0.140 ^ab^	619.650 ± 6.350 ^cd^	58.560 ± 29.110 ^a^
Poultry manure		11.670 ± 0.300 ^a^	5.890 ± 0.350 ^bc^	48.465 ± 0.795 ^c^	9.475 ± 0.025 ^b^	546.900 ± 0.200 ^e^	26.885 ± 1.655 ^b^
Supercompost		9.990 ± 0.170 ^b^	5.410 ± 0.120 ^d^	52.015 ± 1.465 ^bc^	10.055 ± 0.065 ^ab^	603.100 ± 8.700 ^cd^	24.730 ± 0.300 ^b^
Molasses		9.140 ± 0.210 ^c^	5.230 ± 0.040 ^d^	59.635 ± 5.805 ^b^	10.035 ± 0.225 ^ab^	595.350 ± 20.150 ^d^	23.885 ± 1.065 ^b^
Average		10.671	6.169	57.578	10.306	625.171	31.743
CV (%)		9.284	13.156	12.998	6.231	8.192	46.680
ANOVA (*n* = 21)	R^2^	0.938	0.785	0.893	0.622	0.967	0.601
	F	35.534	8.515	19.446	3.836	68.406	3.508
	*p*	<0.001	0.001	<0.001	0.018	<0.001	0.025

Control refers to treatment without any fertilizer application (0 t/ha), B—boron, Cu—copper, Fe—iron, Mn—manganese, Na—sodium, Zn—zinc, R^2^—coefficient of determination, F—variance ratio (F-test), *p*—probability value corresponding to a variance ratio. Mean value ± standard deviation (SD). Values marked with the same letter in a column are not significantly different at 5% (Tukey’s HSD test).

**Table 6:** Soil properties at the experimental site (soil depth 0–30 cm).

pH		CaCO_3_ (%)	Humus (%)	Total N (%)	Al-P_2_O_5_ mg/100 g	Al-K_2_O mg/100 g
KCL	H_2_O					
7.27	8.06	8.64	2.05	0.10	53.74	34.61

**Table 7:** Agrochemical analysis of organic fertilizers.

Organic Manure	N (%)	P_2_O_5_ (%)	K_2_O (%)	Dry Matter (%)	Organic Matter (%)	Organic C (%)
Supercompost	2.28	2.06	0.11	24.54	38.72	10.12
Cattle manure	2.09	1.85	2.06	23.08	71.54	18.74
Sheep manure	1.79	0.97	2.54	26.97	57.73	17.02
Poultry manure	2.50	2.33	2.65	31.36	75.47	16.50
Molasses	2.00	/	5.00	/	/	16.50

The contents of nutrients and organic carbon are expressed as a mass percentage relative to the air-dried sample.

**Table 8:** Applied amounts of fertilizers.

Treatments	Amounts
Control	Without fertilizer application (0 t/ha)
Mineral fertilizer	800 kg/ha NPK (9:12:25) + 288 kg/ha AN (34%N)
Cattle manure	35.2 t/ha
Sheep manure	35.2 t/ha
Poultry manure	21.7 t/ha
Supercompost	30.3 t/ha
Molasses	8.5 t/ha

## Data Availability

Data are contained within the article.

## Abbreviations

The following abbreviations are used in this manuscript:

DW	Dry Weight
TPC	Total Phenolic Content (mg/100 g DW)
TF	Total Flavonoid Content (mg/100 g DW)
DPPH	2,2-Diphenyl-1-picrylhydrazyl radical scavenging assay for antioxidant activity (mg/g DW)
FRAP	Ferric Reducing Antioxidant Power assay (mg Fe^2+^/g DW)
ABTS	2,2′-azino-bis(3-ethylbenzothiazoline-6-sulfonic acid) radical cation decolorization assay for antioxidant activity (mg/g DW)
AA	Antioxidant Activity
PCA	Principal Component Analysis
